# Clinical relevance of dual-energy X-ray absorptiometry (DXA) as a simultaneous evaluation of fatty liver disease and atherosclerosis in patients with type 2 diabetes

**DOI:** 10.1186/s12933-016-0384-7

**Published:** 2016-04-14

**Authors:** Ryotaro Bouchi, Yujiro Nakano, Norihiko Ohara, Takato Takeuchi, Masanori Murakami, Masahiro Asakawa, Yuriko Sasahara, Mitsuyuki Numasawa, Isao Minami, Hajime Izumiyama, Koshi Hashimoto, Takanobu Yoshimoto, Yoshihiro Ogawa

**Affiliations:** Department of Molecular Endocrinology and Metabolism, Graduate School of Medical and Dental Sciences, Tokyo Medical and Dental University, 1-5-45 Yushima, Bunkyo-ku, Tokyo, 113-8510 Japan; Center for Medical Welfare and Liaison Services, Tokyo Medical and Dental University, 1-5-45 Yushima, Bunkyo-ku, Tokyo, 113-8510 Japan; Department of Preemptive Medicine and Metabolism, Graduate School of Medical and Dental Sciences, Tokyo Medical and Dental University, 1-5-45 Yushima, Bunkyo-ku, Tokyo, 113-8510 Japan; Japan Agency for Medical Research and Development, CREST, 1-7-1 Otemachi, Chiyoda-ku, Tokyo, 100-0004 Japan

**Keywords:** Andoroid-to-gynoid ratio, Visceral adiposity, Fatty liver, Atherosclerosis, Type 2 diabetes

## Abstract

**Background:**

Whole body dual-energy X-ray absorptiometry (DXA) can simultaneously measure both regional fat and non-fat mass. Android-to-gynoid (A/G) ratio measured by DXA has been reported to be associated with cardiovascular risks and visceral adiposity; however, little is known regarding its relationship with fatty liver disease and atherosclerosis among patients with diabetes. This study was designed to investigate the association of android and gynoid fat mass measured by DXA with fatty liver disease and atherosclerosis in patients with type 2 diabetes.

**Methods:**

This is a cross-sectional study of 259 patients with type 2 diabetes (mean age 64 ± 13 years; 40.2 % female). Android and gynoid fat mass (kg) were measured by DXA. Skeletal muscle index (SMI) was calculated as appendicular non-fat mass (kg) divided by height (m^2^). Visceral fat area (VFA, cm^2^), subcutaneous fat area (SFA, cm^2^), and liver attenuation index (LAI) were assessed by abdominal computed tomography. Intima media thickness (IMT, mm) in common carotid arteries was determined by carotid ultrasonography.

**Results:**

A/G ratio was significantly correlated with VFA (r = 0.72, p < 0.001), SFA (r = 0.32, p < 0.001) and LAI (r = −0.26, p < 0.001). A/G ratio (standardized β −0.223, p = 0.002) as well as VFA (standardized β −0.226, p = 0.001) were significantly associated with LAI in the univariate model. A/G ratio remained to be significantly associated with LAI (standardized β −0.224, p = 0.005) after adjusting for covariates including body mass index and transaminases. Among patients with low SMI (SMI < 7.0 in male and < 5.4 in female), A/G ratio was significantly associated with carotid IMT in the multivariate model (standardized β 0.408, p = 0.014).

**Conclusions:**

DXA can be used to simultaneously estimate the risks for both fatty liver disease and atherosclerosis in patients with type 2 diabetes.

## Background

Obesity, especially visceral obesity, has been reported to be associated with insulin resistance, dyslipidemia, and hypertension, thus increasing the risk for cardiovascular disease (CVD) [1–4]. Abdominal visceral fat has been strongly associated with cardiovascular risks [5, 6]. However, direct measure of visceral fat is limited due to high cost of imaging procedures such as computed tomography (CT) or magnetic resonance imaging (MRI). Dual-energy X-ray absorptiometry (DXA) is the one that can estimate the risk for visceral fat accumulation with low-cost, relatively quick procedure, and less exposure to ionizing radiation, compared with CT [7, 8]. The clinical utility of DXA is the following; (1) it can measure regional fat mass, compared with anthropological methods such as body mass index (BMI), (2) fat-free mass, mainly reflecting skeletal muscle mass, can be estimated by DXA both in Caucasians and Asians [9–11], leading to the simultaneous evaluation of fat and muscle mass. It is reported that regional fat mass such as android is associated with cardio-metabolic risks [12, 13] and android-to-gynoid ratio (A/G ratio), reflecting visceral fat accumulation, is associated with insulin resistance [14].

Non-alcoholic fatty liver disease (NAFLD) has recently been thought to be a hepatic manifestation of metabolic syndrome [15, 16] and patients with NAFLD are at increased risk for diabetes and CVD [17, 18]. Thus, it is important to measure hepatic fat accumulation for evaluating cardio-metabolic risks as well as the risk for the progression of liver disease; however, the use of CT or MRI is limited due to the high cost and low feasibility. It is also uncertain whether regional fat mass and indices such as A/G ratio measured by DXA could estimate the risk for NAFLD.

CVD is a leading cause of death worldwide. In order to estimate the risk for CVD events, carotid intima media thickness (CIMT) is thought to be a good marker and is associated with incident CVD [19]. Regarding the association of visceral adiposity and CVD, it has been reported that visceral fat mass is significantly associated with incident CVD [20]. Conversely, several previous studies have shown that peripheral subcutaneous fat may represent a “metabolic sink” for the storage of excess energy and may act against metabolic alterations and atherosclerosis [21, 22]. We recently reported that visceral adiposity is associated with arterial stiffness regardless of BMI [23] and visceral-to-subcutaneous fat ratio by abdominal CT is closely associated with CIMT among patients with type 2 diabetes [24]. Others also reported that subcutaneous abdominal fat is inversely associated with carotid atherosclerosis in patients with type 2 diabetes [25]. In addition to visceral and subcutaneous adiposities, skeletal muscle mass is another important determinant of cardio-metabolic alterations and atherosclerosis. Reduced skeletal muscle mass and muscle power (sarcopenia) and increased visceral adiposity are synergistically associated with atherosclerosis as well as diabetes and fatty liver disease [26–28]. These studies suggest the importance of evaluating both visceral and subcutaneous adiposities and muscle mass for the assessment of atherosclerosis.

Considering the potential of DXA in that regional fat and muscle mass can be simultaneously determined, this study was designed to investigate whether DXA could be valuable to concurrently estimate risks for both NAFLD and atherosclerosis in patients with type 2 diabetes.

## Methods

### Subjects

Patients with type 2 diabetes with 20 years of age or older, who were diagnosed as diabetes according to the criteria of the Japan Diabetes Society [29], regularly visited to Tokyo Medical and Dental University Hospital for the purpose of glycemic control, and had undergone the whole body DXA for the evaluation of A/G ratio between July 1, 2012 and December 31, 2015 participated in this study. Patients with alcohol consumption ≥20 g/day in female and 30 g/day in male, severe renal impairment [estimated glomerular filtration rate (eGFR) <15 ml/min/1.73 m^2^ or undergoing renal replacement therapy], pregnant women, those with infectious or malignant diseases, and those without measurement of both visceral fat area (VFA), subcutaneous fat area (SFA) and hepatic fat accumulation [liver-spleen-attenuation index (LAI)] by abdominal CT were excluded. We also excluded patients who had received hepatotoxic drugs including glucocorticoids, tamoxifen, amiodarone, sodium valproate, and methotrexate, and those with other causes of liver diseases such as viral hepatitis (hepatitis B virus/hepatitis C virus) and autoimmune liver diseases. In addition, we excluded patients who had a history of gastrectomy, bariatric or intestinal surgery, malabsorption, those with severe heart failure, those with overt endocrine abnormalities such as hyperthyroidism, those with eating disorder, and those treated with estrogens or anti-psychotics were also excluded, because these diseases or drugs could affect body fat and muscle composition including hepatic fat accumulation. This study complies with the principles laid by Declaration of Helsinki and has been approved by the ethical committee of Tokyo Medical and Dental University (No. M2015-508).

### Clinical and biochemical analysis

Standardized questionnaires were used to obtain information on smoking, medication and past history. Smoking history was classified as either current smoker or non-smoker. CVD was defined as the presence of a previous stroke, myocardial infarction, coronary revascularization procedure. Information regarding diabetic retinopathy was obtained using medical records and the prevalence of proliferative diabetic retinopathy was determined. HbA1c was measured by the latex agglutination method. HbA1c levels were expressed in accordance with the National Glycohemoglobin Standardization Programs recommended by the Japanese Diabetes Society [29]. Systolic and diastolic blood pressures (SBP and DBP) were measured in the sitting position after at least 5 min rest, using an electronic sphyngomanometer (ES-H55, Terumo Inc., Tokyo, Japan). Urinary albumin and creatinine excretion were measured by the turbidimetric immunoassay and enzymatic method, respectively, in a spot urine collection. GFR was estimated using the following equation for the Japanese, as proposed by the Japanese Society of Nephrology [30]; GFR = 194 X SCr^−1.094^ X age^−0.287^ [(if female) X 0.739], where SCr stands for serum creatinine in mg/dl, measured by an enzymatic method. Coefficient of variation of R–R intervals (CV-RR) was used for the assessment of diabetic neuropathy. BMI was calculated as weight divided by the square of height (kg/m^2^).

### Body composition measured by DXA

**Fig. 1:**
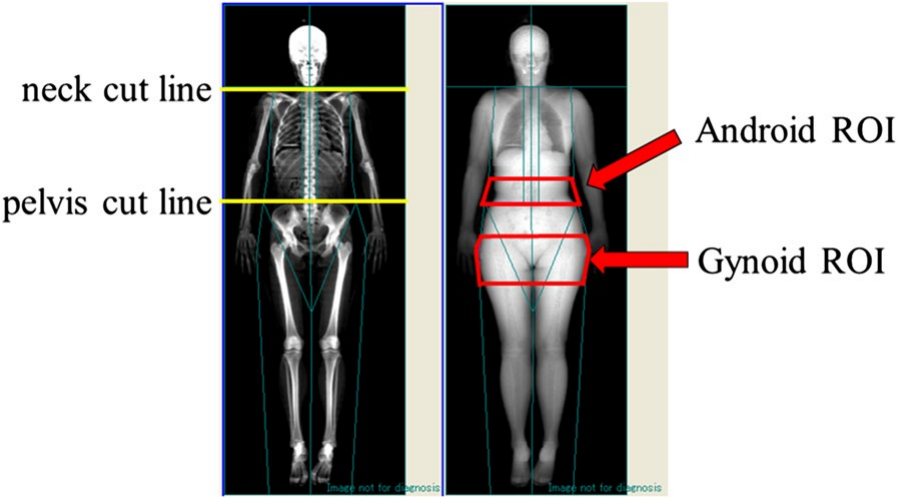
Regional body composition measurement by dual-energy X-ray absorptiometry (DXA). Regions of interest (ROI) in android and gynoid are shown in the *right panel*

Regional fat mass was measured by the whole body DXA (Lunar iDXA, GE Healthcare, Madison, WI). The scans were done by certified radiological technologists who were blinded to the patient characteristics and laboratory data. The imaging machine was calibrated everyday according to the manufacturer’s recommendations. Total and regional scans were taken and two regions (android and gynoid) were considered of interest in this study. The regions of interest (ROI) for regional body composition were defined as previously reported [31]. Briefly, ROI of android was defined as the region from pelvis cut (lower boundary) to above the pelvis cut by 20 % of the distance from pelvis cut line to neck cut line (upper boundary). ROI of gynoid was defined as the region among the 2× Android height, beginning at a distance of 1.5× Android height below pelvis cut (Fig. 1). A/G ratio was the ratio of android (kg) divided by gynoid (kg). Skeletal muscle index (SMI) was calculated as the following: SMI = (fat-free mass in upper and lower extremities, kg) divided by height (m^2^). According to the criteria of sarcopenia in Asia [13], Patients were divided into the following two groups; patients with high SMI (SMI ≥ 7.0 in male and ≥5.4 in female) and those with low SMI (SMI < 7.0 in male and <5.4 in female).

### Quantification of abdominal adiposity, hepatic fat accumulation, and atherosclerosis

VFA and SFA were measured at the level of umbilicus by abdominal CT examination (Aquilion PRIME, Toshiba Medical Systems, Tochigi, Japan). As well as the whole body DXA, the scans were done by certified radiological technologists who were blinded to the patient characteristics and laboratory data and the imaging machine was calibrated everyday according to the manufacturer’s recommendations. Hepatic fat accumulation was determined by LAI in the CT examination, as described previously [32]. Briefly, hepatic and splenic attenuation values were measured on non-contrast CT scans by using eight circular ROI cursors with a diameter of 1.5 cm in the liver and 3 in the spleen. In the liver, four ROIs were located in each of the right anterior, right posterior, left medial, and left lateral segments. In this study, the LAI was evaluated for its efficacy as a marker for steatosis in the liver. Calculation of LAI was as follows: Average attenuation value of liver (eight points) divided by average attenuation value of spleen (three points). Atherosclerosis was assessed by CIMT using an echotomographic system (Aplio XG SSA790A, Toshiba Medical Systems, Tochigi, Japan) with a 7.5-MHz linear transducer, as reported previously [24].

### Statistical analysis

Statistical analysis was performed using programs available in the SPSS version 21.0 statistical package (IBM Corp. Released 2012. IBM SPSS Statistics for Windows, Version 21.0. Armonk, NY: IBM Corp.). Data are presented as mean ± standard deviation (SD), median with interquartile range (IQR), or percent as appropriate according to data distribution. Normality was tested by the Kolmogorov–Smirnov test. Differences between male and female patients were tested with a t test or Mann–Whitney U test (continuous variables) or Chi square test (categorical variables). Pearson product-moment correlation analysis was used to investigate the correlation of A/G ratio with measures for adiposity and LAI. Linear regression analyses with a stepwise procedure were used to assess the cross-sectional association of A/G ratio with LAI and CIMT. We determined the linear relationship and multicollinearity for regression assumptions. We removed one variable if a strong correlation (coefficient of correlation >0.8) was observed between the two independent variables. In order to check the multicollinearity, we evaluated variance infiltration factors. If multicollinearity was found in the data, one variable was removed from the multivariate regression analysis. The following covariates were incorporated into the multivariate analysis; duration of diabetes, smoking status, BMI, SBP, triglycerides (TGs), high-density lipoprotein (HDL) cholesterol, alanine aminotransferase (ALT), HbA1c, eGFR, and the use of insulin. Age and gender were forced into the model. The interaction between A/G ratio and SMI was also investigated where CIMT was used for a dependent variable. Differences were considered to be statistically significant at p value less than 0.05.

## Results

### Clinical characteristics

A total of 259 Japanese patients with type 2 diabetes (mean age 64 ± 12 years; 40.2 % female) were enrolled in this study. Table 1 shows the clinical characteristics by gender. Male patients exhibited significantly higher TGs, uric acid, ALT and glutamyl transpeptidase (γ-GTP), and lower HDL cholesterol levels than female patients. There were no significant differences in age, BMI, glycemic control, blood pressure, low-density lipoprotein (LDL) cholesterol, aspartate transaminase (AST), LAI, and prevalence of diabetic microvascular complications including retinopathy and nephropathy. SMI and VFA were significantly higher and android tended to be higher in male than those in female; by contrast, percentage of body fat, gynoid and SFA were significantly lower in male than those in female. Eventually, male patients had significantly higher A/G ratio and V/S ratio than female.

**Table 1:** Clinical characteristics of patients with type 2 diabetes by gender

	Male (N = 155)	Female (N = 104)	p value
Age (years)	63 ± 12	64 ± 13	0.570
Body mass index (kg/m^2^)	25.1 ± 4.1	24.2 ± 4.4	0.128
HbA1c (%)	7.2 ± 1.4	7.1 ± 1.5	0.600
Duration of diabetes (years)	7 (2–16)	7 (3–10)	0.958
Current smoker (%)	21	3	0.023
Proliferative diabetic retinopathy (%)	8	10	0.423
Insulin (%)	30	36	0.358
Systolic blood pressure (mmHg)	129 ± 14	124 ± 15	0.442
Diastolic blood pressure (mmHg)	77 ± 13	71 ± 15	0.338
Uninary ACR (mg/g)	33 (20–148)	26 (13–54)	0.209
eGFR (ml/min/1.73 m^2^)	75 ± 12	69 ± 13	<0.001
TG (mmol/l)	1.48 (1.30–1.71)	1.22 (1.02–1.47)	0.045
HDL cholesterol (mmol/l)	1.45 ± 0.38	1.60 ± 0.47	0.002
LDL cholesterol (mmol/l)	2.64 (2.19–3.17)	2.69 (2.17–3.72)	0.308
Uric acid (μmol/l)	333 ± 77	286 ± 65	<0.001
AST (U/l)	25 (20–34)	22 (18–28)	0.402
ALT (U/l)	26 (16–45)	20 (15–33)	0.047
γ-GTP (U/l)	42 (31–53)	30 (20–40)	<0.001
Body fat (%)	30.8 ± 6.8	38.7 ± 7.2	<0.001
Skeletal muscle index	7.2 ± 1.1	5.8 ± 1.0	<0.001
Android (kg)	2.1 (1.1–3.3)	1.9 (1.5–2.9)	0.098
Gynoid (kg)	2.7 (1.9–3.8)	3.3 (2.5–4.1)	0.006
A/G ratio	0.74 ± 0.18	0.59 ± 0.19	<0.001
Visceral fat area (cm^2^)	153 (95–228)	126 (104–169)	0.002
Subcutaneous fat area (cm^2^)	141 (74–231)	194 (150–252)	<0.001
V/S ratio	1.14 ± 0.50	0.65 ± 0.32	<0.001
Liver attenuation index	1.08 ± 0.22	1.17 ± 0.29	0.303

*ACR* albumin-to-creatinine ratio, *A/G* android-to-gynoid, *ALT* alanine aminotransferase, *AST* Aspartate aminotransferase, *eGFR* estimated glomerular filtration rate, *γ-GTP* γ-glutamyl transpeptidase, *HDL* high-density lipoprotein, *LAI* liver attenuation index, *LDL* low-density lipoprotein, *V/S* visceral-to-subcutaneous fat

### Correlation of A/G ratio with markers for body composition

**Fig. 2:**
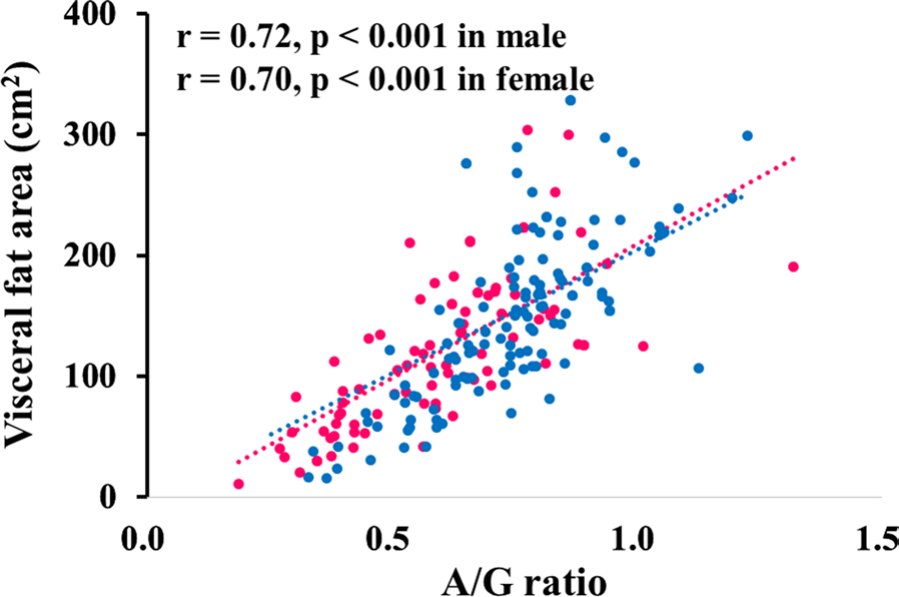
Correlation between android-to-gynoid (A/G) ratio and visceral fata area (VFA) in patients with type 2 diabetes. *Blue* and *pink circles* indicate male and female patients. p values by Pearson product-moment correlation analysis

As shown in Table 1, there were definite differences in body fat composition between male and female; thus, we investigated the correlation between A/G ratio and VFA by gender. Figure 2 shows the correlation between A/G ratio and VFA among the patients. A/G ratio was significantly and strongly correlated with VFA both in male (r = 0.72, p < 0.001) and female (r = 0.70, p < 0.001). Compared with the relationship between A/G ratio and VFA, the correlations of A/G ratio with other markers for body composition were weak (r = 0.32 and p < 0.001 for SFA, r = 0.26 and p = 0.001 for percentage of body fat, r = 0.52 and p < 0.001 for BMI).

### Association of A/G ratio and VFA with hepatic fat accumulation

**Fig. 3:**
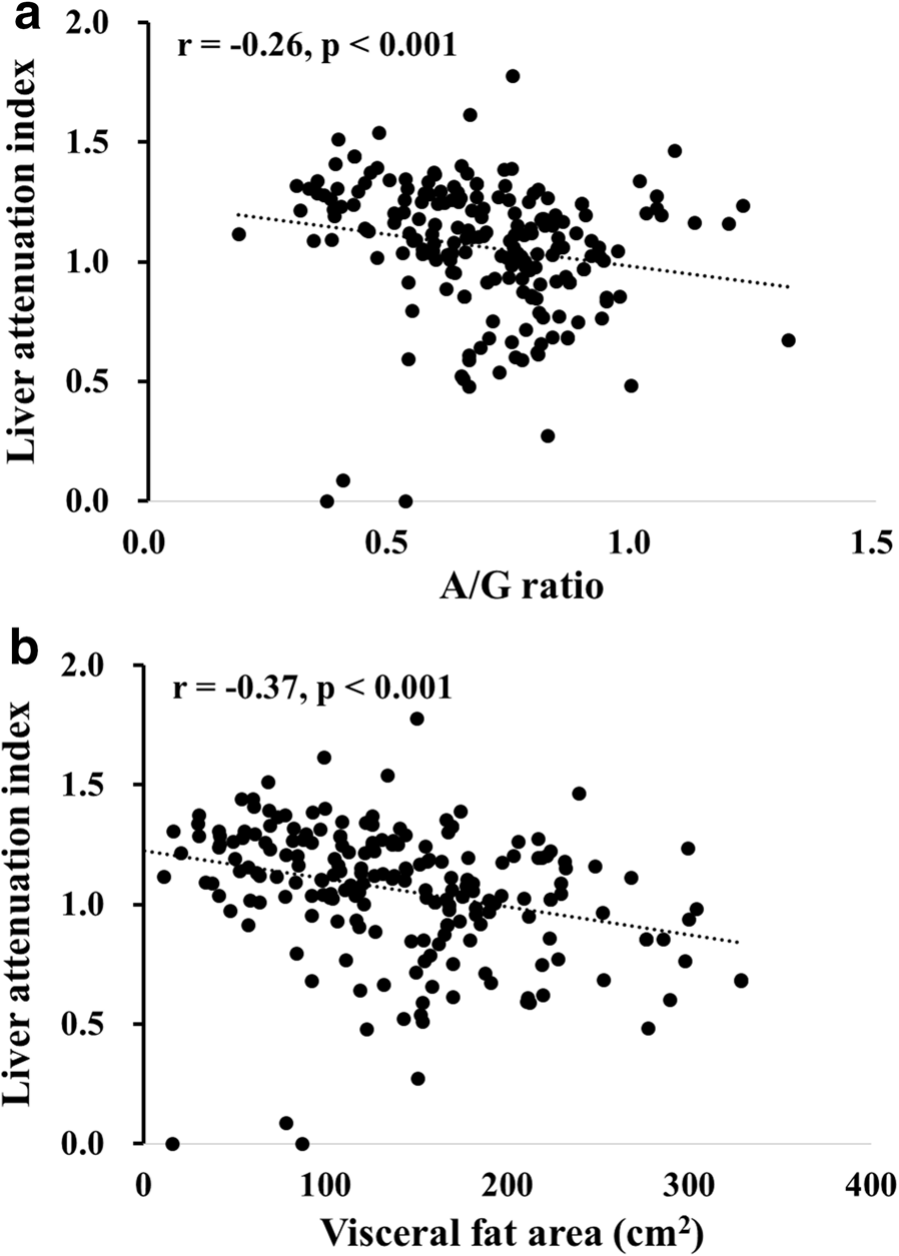
Correlation of android-to-gynoid (A/G) ratio (**a**) and visceral fata area (VFA) (**b**) with liver attenuation index (LAI) in patients with type 2 diabetes. p values by Pearson product-moment correlation analysis

Figure 3 shows the correlation of A/G ratio (Fig. 3a) and VFA (Fig. 3b) with LAI among the patients. A/G ratio was modestly correlated with LAI (r = −0.26, p < 0.001) and its relationship was almost identical to the relationship between VFA and LAI (r = −0.37, p < 0.001). In the univariate linear regression model as shown in Table 2, A/G ratio was significantly associated with LAI (standardized β −0.223, p = 0.002). A/G ratio remained to be significantly associated with LAI after adjustment for covariates including ALT and HDL cholesterol (multivariate model 1). Further adjustment of BMI did not attenuate the significant association between A/G ratio and LAI (Multivariate model 2).

**Table 2:** Linear regression analysis to determine the relationship between A/G ratio and LAI in patients with type 2 diabetes

	Standardized β	p value
Univariate (Adjusted R^2^ = 0.045)		
A/G ratio	−0.223	0.002
Multivariate model 1 (Adjusted R^2^ = 0.076)		
A/G ratio	−0.2240	0.005
ALT	−0.196	0.014
HDL cholesterol	−0.131	0.096
Multivariate model 2 (Adjusted R^2^ = 0.096)		
A/G ratio	−0.142	0.043
ALT	−0.182	0.016
HDL cholesterol	−0.151	0.044
Body mass index	−0.196	0.017

*A/G* android-to-gynoid, *ALT* alanine aminotransferase, *HDL* high-density lipoprotein, *LAI* liver attenuation index

### Cardiovascular risk prediction by A/G ratio and VFA

We next investigated whether A/G ratio could have a similar ability to VFA in prediction of cardiovascular risks such as glycemic, lipid and blood pressure control (Table 3). Other than albuminuria, the correlations of A/G ratio with cardiovascular risk factors (HbA1c, TG, HDL cholesterol, SBP, uric acid, and ALT) were almost similar to VFA in the univariate model. Regarding the association of A/G ratio and VFA with insulin resistance, we used the TG/HDL cholesterol ratio as a surrogate marker for insulin resistance. A/G ratio was significantly correlated with TG/HDL cholesterol ratio as well as VFA.

**Table 3:** Univariate correlation of A/G ratio and VFA with cardio-metabolic markers in patients with type 2 diabetes

	A/G ratio	p value	VFA	p value
HbA1c	0.149	0.027	0.193	0.005
Triglycerides	0.236	<0.001	0.239	<0.001
HDL cholesterol	−0.192	0.004	−0.152	0.026
TG/HDL cholesterol ratio	0.234	<0.001	0.211	<0.001
Age	−0.127	0.054	−0.137	0.044
Systolic blood pressure	0.346	<0.001	0.231	<0.001
Uric acid	0.305	<0.001	0.316	<0.001
ALT	0.207	0.002	0.336	<0.001
Log urinary ACR	0.052	0.463	0.147	0.044

*ACR* albumin-to-creatinine ratio, *A/G* android-to-gynoid, *ALT* alanine aminotransferase, *HDL* high-density lipoprotein, *TG* triglycerides, *VFA* visceral fat area

### Association of A/G ratio and muscle mass with carotid atherosclerosis

Among the total subjects, 105 patients underwent carotid ultrasonography for the assessment of atherosclerosis (CIMT median 0.85 mm, IQR 0.71–1.00 mm). The interaction between A/G ratio and SMI was observed in the whole cohort (p = 0.045 in the linear regression analysis). Thus, we investigated the association of A/G ratio with CIMT in patients with low SMI and those with high SMI (Table 4). Among patients with low SMI (N = 65), A/G ratio was significantly associated with CIMT in the univariate model. The statistical significance between A/G ratio and CIMT remained unchanged after adjusting for age and gender. In the multivariate linear regression analysis, A/G ratio and eGFR were significant predictors for the risk of carotid atherosclerosis. By contrast, among patients with high SMI (N = 40), no significant association between A/G ratio and CIMT was observed in both univariate and multivariate models.

**Table 4:** Association of A/G ratio with carotid atherosclerosis in patients with type 2 diabetes according to skeletal muscle mass

	High SMI (N = 65)		Low SMI (N = 40)	
	SMI ≥ 7.0 in male		SMI < 7.0 in male	
	SMI ≥ 5.4 in female		SMI < 5.4 in female	
Univariate model	(Adjusted R^2^ = 0.019)		(Adjusted R^2^ = 0.214)	
	Standardized β	p value	Standardized β	p value
A/G ratio	−0.017	0.903	0.485	0.002
Age- and gender-adjusted model	(Adjusted R^2^ = 0.189)		(Adjusted R^2^ = 0.217)	
	Standardized β	p value	Standardized β	p value
A/G ratio	−0.025	0.867	0.405	0.014
Age	0.462	0.001	0.168	0.269
Gender	0.199	0.175	0.157	0.308
Multivariate model	(Adjusted R^2^ = 0.336)		(Adjusted R^2^ = 0.220)	
	Standardized β	p value	Standardized β	p value
A/G ratio	0.045	0.765	0.408	0.010
Age	0.487	<0.001	0.175	0.252
Gender	0.026	0.859	0.150	0.342
Systolic blood pressure	0.303	0.023	0.310	0.042
Urinary ACR	0.306	0.016	NA	
HDL cholesterol	−0.309	0.025	NA	

*ACR* albumin-to-creatinine ratio, *A/G* android-to-gynoid, *ALT* alanine aminotransferase, *HDL* high-density lipoprotein, *VFA* visceral fat area

## Discussion

Our study demonstrates that A/G ratio, measured by DXA, is correlated with visceral fat accumulation measured by CT and can estimate the risks for both NAFLD and atherosclerosis in patients with type 2 diabetes, suggesting that noninvasive and readily available measure of regional body fat (android and gynoid) by DXA could deserve to be considered for evaluating both NAFLD and atherosclerosis as well as direct measure of body fat by CT or MRI. We also revealed that DXA has the advantage of being able to simultaneously determine the high-risk group of atherosclerosis with high visceral adiposity and low muscle mass, compared with CT or MRI. The association between NAFLD and atherosclerosis has recently attracted attention over the years because NAFLD is thought to be a hepatic manifestation of metabolic syndrome [15, 16] and is associated with progression of atherosclerosis [33, 34] and incident CVD [18]. In addition, a recent large-scale observational study demonstrates that NAFLD is associated with increased CIMT in patients with type 2 diabetes and insulin resistance [35]. Thus, we sought to investigate the association of regional body fat and non-fat mass measured by the whole body DXA with the risks for both NAFLD and atherosclerosis among patients with type 2 diabetes.

### Regional body fat measured by DXA and visceral adiposity measured by CT

First, we found a significant correlation between A/G ratio and VFA in this study. Visceral adiposity is thought to be located in the upstream of metabolic syndrome and its strong association with cardio-metabolic risks [6, 36, 37] and incident CVD [20] has been widely reported. DXA can perform for the assessment of visceral fat as shown by the good correlation of android measured by DXA with direct measure of visceral fat measured by CT [38]. The finding in this study is consistent with the previous study [38] in that DXA can be used for the assessment of visceral adiposity with low cost, and high safety and availability, compared with CT or MRI. Furthermore, in prediction of cardio-metabolic risks, the utility of DXA has recently been reported [39]. Actually, the good correlations of A/G ratio with markers for cardio-metabolic risks such as blood pressure, lipid profiles in this study well support the performance of DXA in prediction of cardio-metabolic risks.

### Association between A/G ratio and insulin resistance

Visceral obesity is accompanied by increased production of free fatty acids, which are transported through the portal circulation to the liver and further to peripheral tissues, especially to the skeletal muscle, leading to insulin resistance of peripheral tissues. Thus, we determined the association between A/G ratio and TG/HDL cholesterol ratio as the surrogate marker for insulin resistance [40] and found a significant correlation between A/G ratio and TG/HDL cholesterol ratio. These observations suggest that A/G ratio may reflect the peripheral insulin resistance in addition to the utility of DXA for estimating visceral fat accumulation.

### Association between A/G ratio and NAFLD

It is important to estimate the risk for fatty liver disease among diabetic patients because diabetes is a strong predictor for progression of NAFLD (liver cirrhosis and hepatocellular carcinoma) [41] and hepatic fat accumulation has been reported to be associated with incident CVD in patients with type 2 diabetes [18]. In this study, we for the first time revealed that A/G ratio is significantly associated with hepatic fat accumulation in patients with diabetes. In addition, we recently reported the association of VFA and BMI with hepatic fat accumulation in patients with type 2 diabetes, showing that patients with high VFA and normal BMI were at significantly increased risk for prevalent fatty liver disease as well as those with high VFA and high BMI [32]. Thus, we investigated whether BMI could attenuate the association between A/G ratio and hepatic fat accumulation (multivariate model 2 in Table 2) and found that A/G ratio remained to be significantly associated with hepatic fat accumulation regardless of BMI. The finding in this study expands the predictive ability of DXA from visceral adiposity and cardio-metabolic risks to hepatic fat accumulation.

### Performance of DXA for prediction of the risk for atherosclerosis

In this study, we found an interaction between A/G ratio and SMI on the risk for atherosclerosis. Thus, we divided patients into those with high and low SMI and performed linear regression analyses to investigate the association between A/G ratio and CIMT. Finally, we revealed that A/G ratio is a significant determinant of atherosclerosis in patients with reduced skeletal muscle; whereas, its relationship with CIMT was not significant in patients with high skeletal muscle mass. A previous study demonstrated that sarcopenic obesity, defined as reduced skeletal muscle mass and power with increased (visceral) obesity, is associated with CVD events [42] and arterial stiffness [43]. Thus, the finding in this study suggests that high A/G ratio with low SMI may reflect sarcopenic obesity, resulting in increased risk for carotid atherosclerosis.

### Clinical implications of high A/G ratio with NAFLD and atherosclerosis

NAFLD is more closely associated with coronary calcification than abdominal obesity [44]. Indeed, the severity of NAFLD has been associated with coronary calcification [45]. NAFLD patients with high γ-GTP levels have increased risk for CVD [46]. Among patients with type 2 diabetes, cardiac structure and function have been reported to be altered in those with NAFLD even without overt CVD [47]. In addition, NAFLD is associated with low-grade inflammation, oxidative stress, and changes in gut microbiota [48, 49]. Thus, NAFLD is thought to be a hepatic component of metabolic syndrome and also share a common pathophysiology with CVD. In this study, patients with high A/G ratio were at increased risk for both NAFLD and atherosclerosis, suggesting that body fat distribution may be one of the candidates that can account for the link between NAFLD and atherosclerosis. Further studies are needed to investigate whether changes in body fat distribution could be associated with the improvement of both NAFLD and atherosclerosis.

## Limitations

This study has a couple of limitations that should be mentioned. First, the study design was cross-sectional, which might preclude casual correlation. Second, population in this study was ethnically and socially homogeneous, because this study was hospital-based; therefore, generalization of our findings might be limited. Third, we had no information on muscle strength such as handgrip power. Thus we were unable to investigate the combined effect of sarcopenia and visceral adiposity on atherosclerosis, although we revealed the significant association between increased visceral adiposity with reduced muscle mass and carotid IMT. Fourth, we used TG/HDL cholesterol ratio as the surrogate marker for insulin resistance. Thus, further studies are needed to precisely investigate the association between regional fat mass by DXA and insulin resistance using indices such as homeostasis model assessment-insulin resistance (HOMA-IR). Finally, it is to be elucidated whether A/G ratio could be associated with hepatic fat accumulation and atherosclerosis in non-diabetic populations.

In summary, our data suggest that DXA is valuable to simultaneously determine increased risks for both hepatic fat accumulation and atherosclerosis in patients with type 2 diabetes.

## Abbreviations

ACR: albumin-to-creatinine ratio
A/G: android-to-gynoid
ALT: alanine aminotransferase
ARB: angiotensin receptor blocker
AST: asparatate aminotransferase
BMI: body mass index
CCB: calcium channel blocker
CI: confidence interval
CIMT: carotid intima media thickness
CT: computed tomography
CVD: cardiovascular disease
CV-RR: coefficient of variation of R–R intervals
DXA: dual X-ray absorptiometry
eGFR: estimated glomerular filtration rate
γ-GTP: glutamyl transpeptidase
HDL: high-density lipoprotein
HOMA-IR: homeostasis model assessment-insulin resistance
LAI: liver-spleen-attenuation index
LDL: low-density lipoprotein
MRI: magnetic resonance imaging
NAFLD: non-alcoholic fatty liver disease
PDR: proliferative diabetic retinopathy
SFA: subcutaneous fat area
SMI: skeletal muscle index
TG: triglyceride
VFA: visceral fat area
